# Remodeling of the *Candida glabrata* cell wall in the gastrointestinal tract affects the gut microbiota and the immune response

**DOI:** 10.1038/s41598-018-21422-w

**Published:** 2018-02-20

**Authors:** Rogatien Charlet, Youri Pruvost, Gael Tumba, Fabian Istel, Daniel Poulain, Karl Kuchler, Boualem Sendid, Samir Jawhara

**Affiliations:** 1 INSERM, U995/Team2, Lille, F-59000 France; 2 University Lille2, U995-LIRIC, Lille Inflammation Research International Center, Lille, F-59000 France; 3 CHU Lille, Service de Parasitologie Mycologie, Pôle de Biologie Pathologie Génétique, Lille, F-59000 France; 4 Medical University Vienna, Max F. Perutz Laboratories, Department of Medical Biochemistry, Vienna, Austria

## Abstract

The gastrointestinal (GI) microbiota acts a natural barrier to the proliferation of opportunistic pathogens. *Candida glabrata* is an opportunistic yeast pathogen that has adapted to colonize all segments of the human GI tract. We observed an increase in *Escherichia coli*, *Enterococcus faecalis*, and *Bacteroides vulgatus* populations, and a decrease in *Lactobacillus johnsonii*, *Bacteroides thetaiotaomicron*, and *Bifidobacterium animalis* in mice with DSS-induced colitis. This reduction was more pronounced for *L. johnsonii* during *C. glabrata* overgrowth. In addition, *C. glabrata* overgrowth increased mouse mortality and inflammatory parameters, and modulated the expression of intestinal receptors and signaling pathways. The *C. glabrata* cell wall underwent various changes during the course of *C. glabrata* colonization, and showed a significant increase in chitin. *C. glabrata* deficient in chitin synthase-3 induced fewer inflammatory parameters than the parental strain during intestinal inflammation. Oral administration of chitin attenuated the impact of colitis, and reduced the number of aerobic bacteria and *C. glabrata* overgrowth, while chitinase-3-like protein-1 increased. This study provides evidence that inflammation of the gut alters the microbial balance and leads to *C. glabrata* cell wall remodeling through an increase in chitin, which is involved in promoting persistence of *C. glabrata* in the gut.

## Introduction

The gastrointestinal (GI) microbiota acts a natural barrier to colonization and proliferation of opportunistic pathogens, decreasing the risk of intestinal infection and disease. Deregulation of the dynamic crosstalk between the microbiota, intestinal epithelial cells and immune cells is critically involved in the development of inflammatory bowel disease (IBD). IBD is a chronic inflammatory disease of the GI tract, which includes Crohn’s disease (CD) and ulcerative colitis (UC)^1^. CD and UC are distinguishable by the location of the inflammation and by the pattern of histologic alterations in the GI tract.

A recent study has revealed that CD patients have significantly higher quantities of fungal species than healthy subjects, and that this is positively correlated with high levels of anti-*Saccharomyces cerevisiae* antibodies (ASCA)^2^. Animal models have played a significant role in increasing our understanding of IBD pathogenesis, especially models of murine colitis^3^. Experimental studies have shown that either *Candida albicans* or *C. glabrata* aggravate intestinal inflammation induced by dextran sulfate sodium (DSS) in mice, and, conversely, that DSS colitis promotes fungal colonization^4,5^.

Like *C. albicans, C. glabrata* is an opportunistic fungal pathogen commonly found in the human GI tract. *C. glabrata* is a particular problem in immunocompromized patients where it can disseminate from the GI tract to cause invasive candidiasis (IC)^6,7^, which is associated with high rates of morbidity and mortality^8,9^.

The fungal cell wall is the predominant site of interaction between the fungus and its host. This cell wall consists of a complex structure of polysaccharides, proteins, and lipids^7^, but its composition is dynamic, responding to changes in the local environment^7,10^. Expansion of the fungal wall during growth involves permanent remodeling of the cell wall polysaccharide network, which is comprised of three major types of polysaccharide: mannans, β-glucans, and chitin. Chitin is a homopolymer of β1,4-N-acetylglucosamine (GlcNAc) and is essential for biological functions in fungi, including cell division^11^, forming the primary septum of all septa, hyphal growth^12^, and virulence^13,14^. Chitin synthesis in *C. glabrata* is carried out by chitin synthases^15^. Deregulation of chitin biosynthesis is a potential mechanism of virulence and resistance to antifungal treatments.

Chitin has been reported to have anti-ulcer^16^, anti-tumor^17^, and anti-inflammatory^18^ properties. Chitin is recognized by different receptors, triggering an immune response. Previous investigations have shown that NOD-2 and TLR-9 recognize chitin and act together to mediate an anti-inflammatory response via secretion of the cytokine, interleukin (IL)-10^19^.

In the present study, we investigated the impact of *C. glabrata* colonization on the gut microbiota diversity in a DSS-induced colitis model, and assessed how the *C. glabrata* cell wall is remodeled in order to persist in the gut environment. We also analyzed the effect of chitin deficiency on *C. glabrata*-host interactions in the DSS mouse model.

## Results

### Measurement of inflammatory parameters

Mice were administered an oral dose of *C. glabrata* WT and were exposed to DSS treatment for 2 weeks in order to induce acute colitis. Mice given *C. glabrata* WT only showed no signs of inflammation or mortality (Supplementary data Fig. 1 and Table 1). In contrast, mice given DSS or *C. glabrata* WT-DSS showed, from day 6, a decrease in body weight and mortality rates of 10% and 20%, respectively. The clinical score for inflammation was significantly higher in *C. glabrata* WT-DSS mice than in the DSS group, starting gradually from day 6, in which the first clinical symptoms of inflammation appeared, including diarrhea and bloody stools (Supplementary data Fig. 1B). The histologic score, which was based on the degree of inflammatory cell infiltration and the amount of tissue damage, was significantly higher in *C. glabrata* WT-DSS mice than in DSS-treated mice (Supplementary data Fig. 1C). Epithelial damage was observed along all parts of the colon mucosa, and leukocyte infiltrates, crypt abscesses and mucosal edema were more common in the colons from *C. glabrata* WT-DSS mice than in those from mice treated with DSS only (Supplementary data Fig. 1D).

### Impact of *C. glabrata* colonization on the gut microbiota

**Figure 1:**
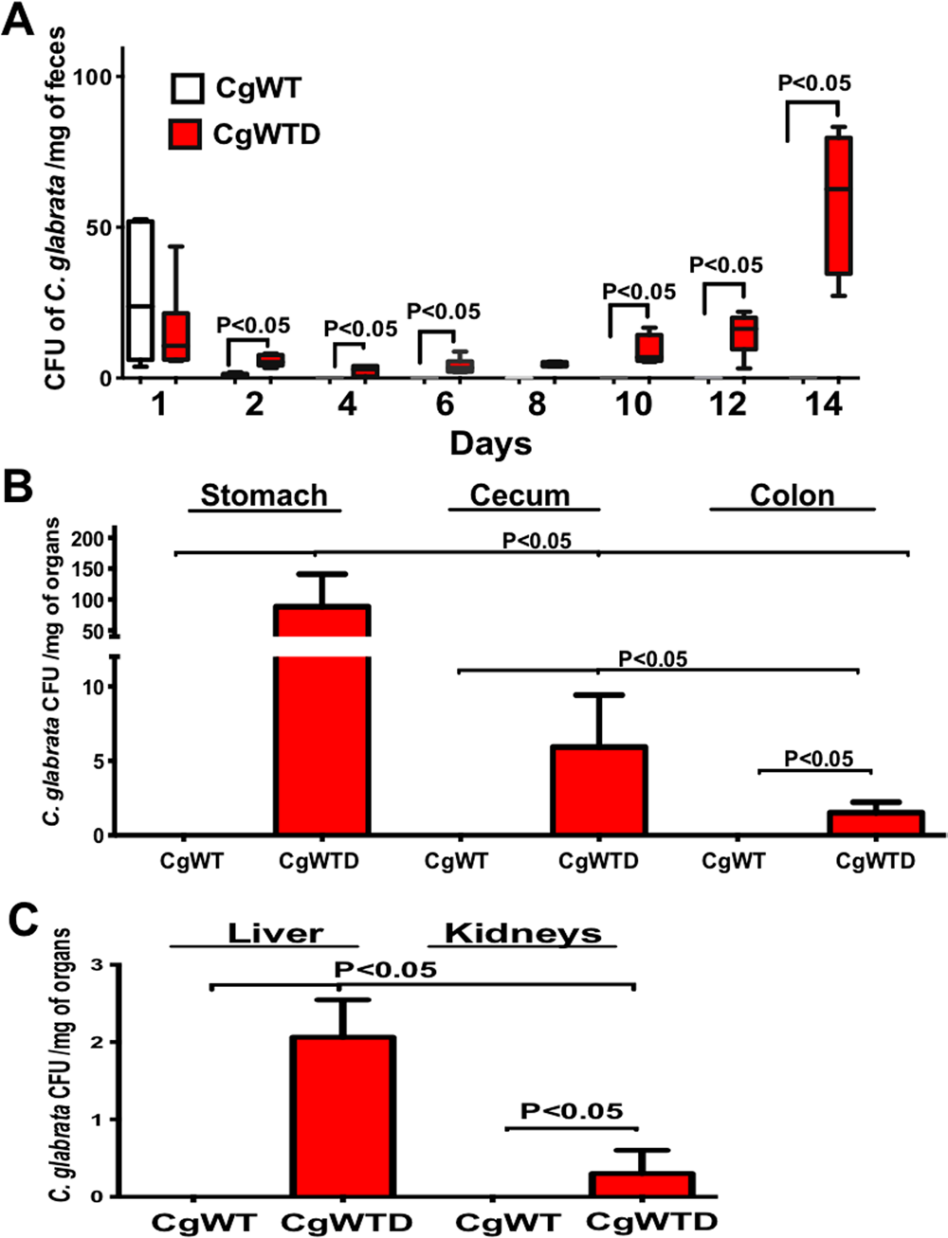
*C. glabrata* colonization in mouse DSS-induced colitis. (**A**) Number of *C. glabrata* colony forming units (CFU) recovered from stools. Data are the mean ± SD of 16 mice per group. (**B**) Number of *C. glabrata* CFU recovered from the stomach, cecum, and colon. Data are the mean ± SD of 20 mice per group (*P* < 0.001). (**C**) Number of *C. glabrata* CFU recovered from the liver and kidneys. Data are the mean ± SD of 16 mice per group (*P* < 0.001).

The number of *C. glabrata* CFUs and the changes in microbiota diversity were determined in freshly collected stool samples from tagged mice, using traditional culture methods based on agar plates (Fig. 1). A high number of *C. glabrata* CFUs was observed in both *C. glabrata* WT and *C. glabrata* WT-DSS groups on day 1 (Fig. 1A). In the absence of DSS, *C. glabrata* was eliminated from the mice from day 2. In contrast, the number of *C. glabrata* CFUs increased from day 2 in the *C. glabrata* WT-DSS group, and remained significantly higher than that in the *C. glabrata* WT group up to day 14 (P < 0.05).

To assess the extent of *C. glabrata* colonization in the GI tract, we assessed the number of yeasts adhering to the stomach, caecum, and colon (Fig. 1B). Significantly higher numbers of viable *C. glabrata* were detected in the stomach, caecum, and colon of *C. glabrata* WT-DSS mice than in mice colonized with *C. glabrata* WT (P < 0.001).

**Figure 2:**
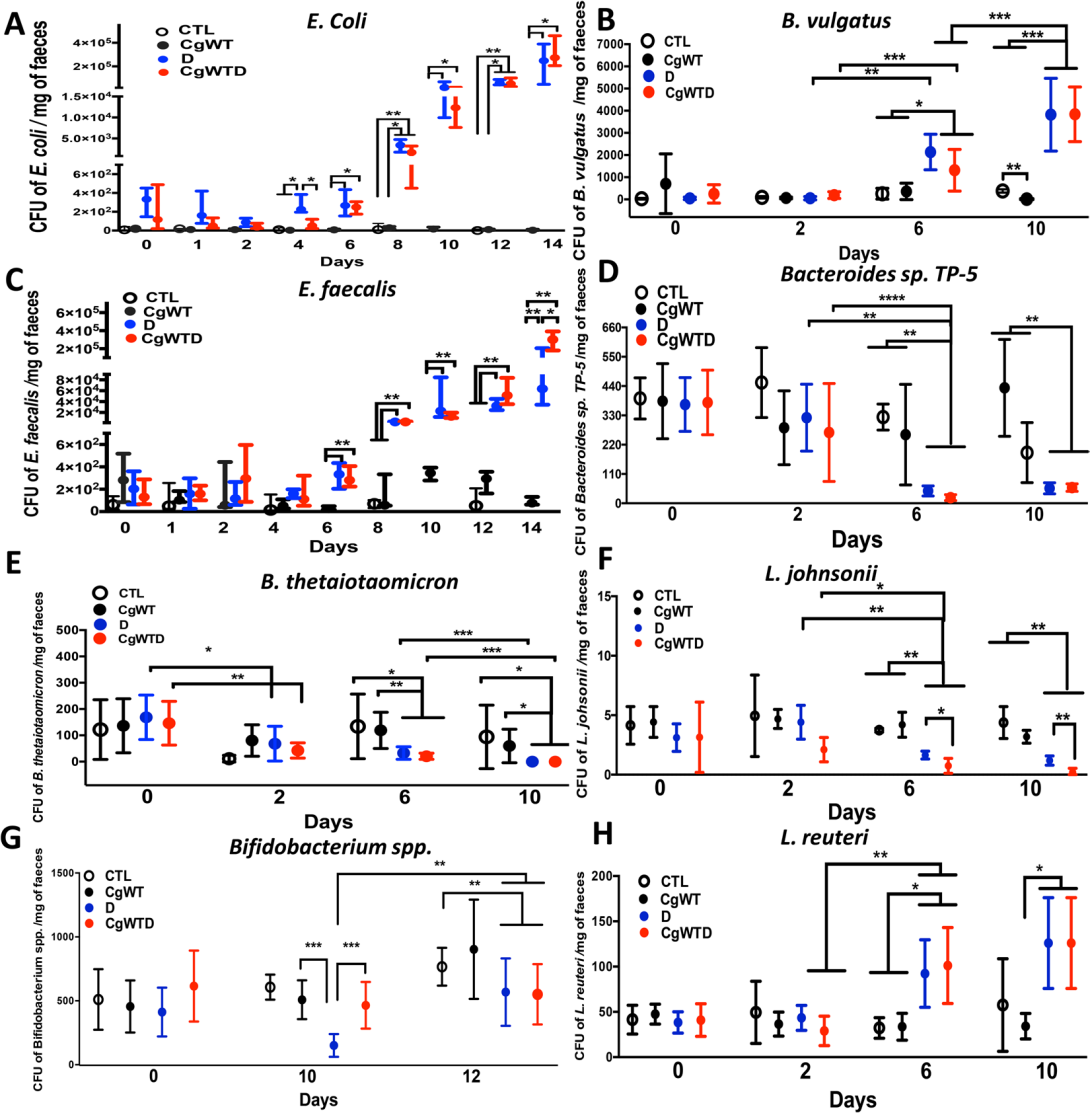
Measurement of viable fecal microorganisms in DSS-induced colitis. The four groups consisted of controls (water), *C. glabrata* alone (*Cg*), DSS alone (D), and *C. glabrata* + DSS (*Cg*D). Data are the mean ± SD of 16 mice per group. For all experiments, stool bacteria were isolated from mice on day 0 before *C. glabrata* challenge and DSS treatment. (**A–H**) Enumeration of *E. coli, B. vulgatus, E. faecalis, Bacteroides* spp*. TP-5, B. thetaiotaomicron, L. johnsonii, Bifidobacterium* spp., and *L. reuteri* CFUs in stool samples. Data are the mean ± SD of 16 mice per group (**P < 0.001, *P < 0.05).

DSS-induced colitis promoted *C. glabrata* dissemination to the liver and kidneys (Fig. 1C). Regarding the microbiota diversity, cultures confirmed that the number of *E. coli* and *E. faecalis* colonies increased significantly from day 6 to day 14 in both the DSS and *C. glabrata* WT-DSS groups when compared to the *C. glabrata* WT or control groups, suggesting that irrespective of *C. glabrata* colonization, DSS-induced colitis promotes an increase in *E. coli* and *E. faecalis* populations in mice (Fig. 2A,C). In contrast to *Bacteroides vulgatus* populations, the number of *B. thetaiotaomicron* and *Bacteroides* sp. *TP5* decreased significantly in both DSS and *C. glabrata* WT-DSS mice (Fig. 2B,D and E). In terms of anaerobic bacteria, the number of *Bifidobacterium spp*., in particular *Bifidobacterium animalis*, and *L. johnsonii* colonies was significantly reduced in both DSS and *C. glabrata* WT-DSS mice, but the reduction in the *L. johnsonii* population was significantly more pronounced in the *C. glabrata* WT-DSS group, suggesting that overgrowth of *C. glabrata* impacts on the *L. johnsonii* population during the development of colitis (Fig. 2F and G). In contrast, the *Lactobacillus reuteri* population revealed unpredictable changes and significant fluctuations over the course of DSS-induced colitis suggesting no association between *C. glabrata*-induced intestinal inflammation and *L. reuteri* levels (Fig. 2H).

### Analysis of cytokines, signaling pathways, and receptor expression

**Figure 3:**
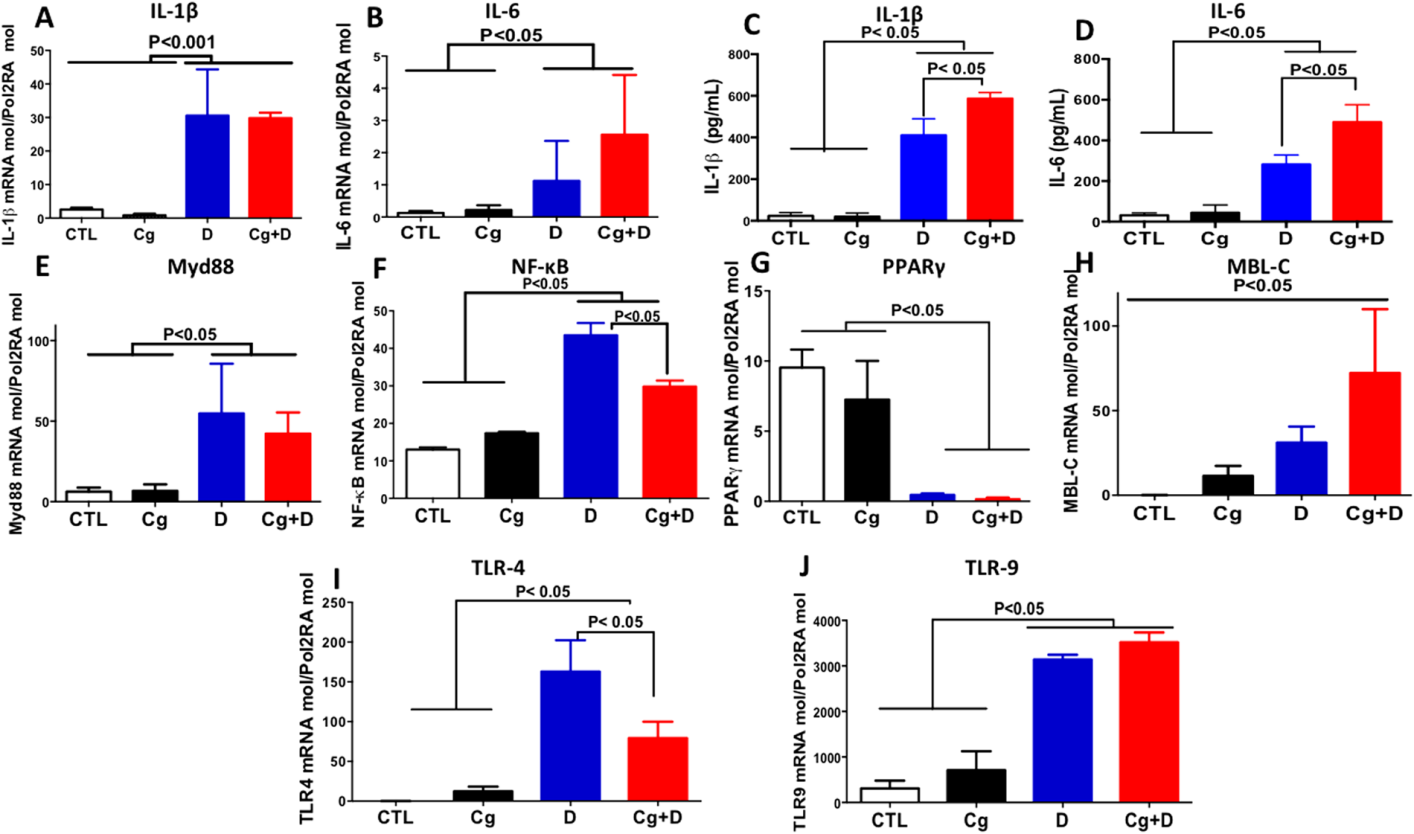
Cytokine and receptor expression in *C. glabrata* wild-type and DSS-induced colitis. (**A** and **B**) Relative expression levels of IL-1β, and IL-6 mRNA in mouse colons. (**C** and **D**) Protein levels of IL-1β and IL-6 in mouse colons. (**F**–**J**) Relative expression levels of Myd88, NF-κB, PPARγ, MBL-C, TLR-4, and TLR-9 mRNA in mouse colons. Data are the mean ± SD of 16 mice per group (*P < 0.05).

To understand the mechanism by which changes in the microbiota modulate the inflammatory mediator responses during colitis and colonization with *C. glabrata*, pro-inflammatory cytokine expression (including IL-1β, and IL-6) was measured in the mice colons. Expression of IL-1β and IL-6 was significantly higher in the colons of *C. glabrata* WT-DSS mice than in DSS mice (Fig. 3A–D). Conversely, expression of these cytokines was significantly lower in the colons of *C. glabrata* WT mice and control groups. In terms of PPARγ and Myd88 expression, there were no significant differences between DSS and *C. glabrata* WT-DSS groups (Fig. 3E,G). To determine the activation/expression of receptors in response to *C. glabrata* sensing and colitis, the expression levels of TLR-4, TLR-9, and MBL-C were examined. DSS-induced colitis significantly increased TLR-4, TLR-9, and MBL-C expression in colon tissues. TLR-9 and MBL-C expression increased significantly in response to both *C. glabrata* and colitis, when compared to mice treated with DSS only (Fig. 3H–J), although TLR-4 expression decreased.

### Remodeling of the *C. glabrata* cell wall after passage through the GI tract

**Figure 4:**
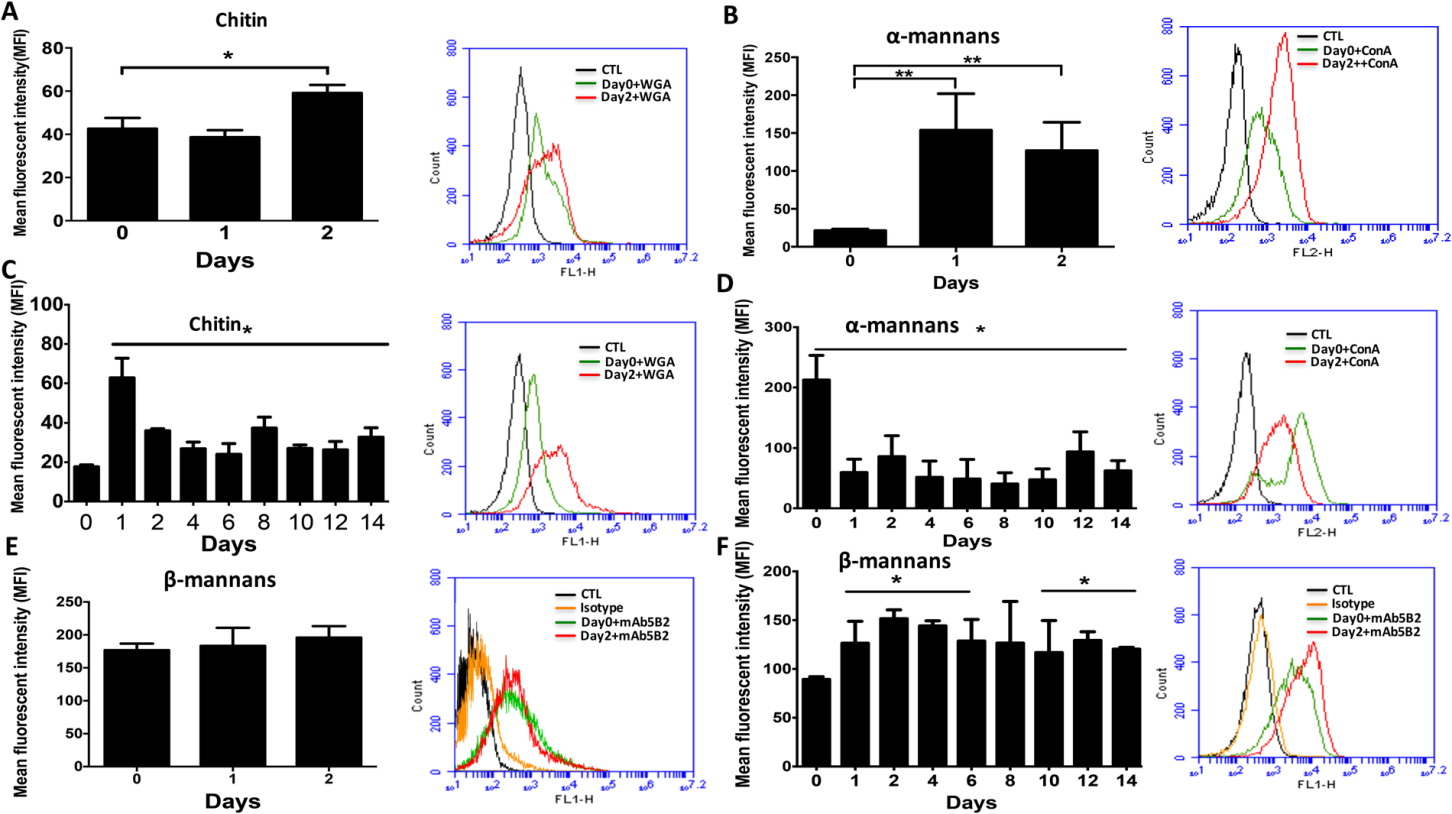
Flow cytometry analysis of the expression of *C. glabrata* cell wall surface glycans after passage through the digestive tract. The expression of cell wall surface glycans was determined in *C. glabrata* using mAbs 5B2 and WGA, and concanavalin A immunofluorescent staining. (**A**,**B** and **E**) *C. glabrata* cell wall surface glycan expression analyzed in mice receiving only *C. glabrata*. (**C**,**D**,**F**) *C. glabrata* cell wall surface glycan expression analyzed in mice receiving *C. glabrata* and DSS. Black and orange peaks (controls) are either fluorescent staining or isotype mAb background fluorescence for each specific mAb or immunofluorescent stain investigated.

To assess whether *C. glabrata* undergoes cell wall remodeling during fungal colonization in the DSS-induced colitis model, we analyzed the cell wall fitness in terms of α-mannans (α-mans), β-mans, and chitin in the stools of tagged mice over a 2 week period. This analysis was carried out using flow cytometry by measuring the median fluorescence intensity (MFI) (Fig. 4A,B and E).

In the absence of DSS, *C. glabrata* was rapidly eliminated from the mouse gut; the fitness of the fungal cell wall was therefore only analyzed over 2 days. A significant increase in α-mans was observed, while the chitin level did not change significantly between day 0 and day 1 (Fig. 4).

In DSS-induced colitis, a significant increase in chitin and β-man levels was observed, while α-mans decreased significantly (Fig. 4C,D,F). Remodeling of the fungal cell wall was also assessed by confocal microscopy and was consistent with the flow cytometry analysis, indicating that both β-mans and chitin are involved in promoting persistence of *C. glabrata* in the gut.

### Impact of chitin deficiency on *C. glabrata* virulence in the mouse colitis model

**Figure 5:**
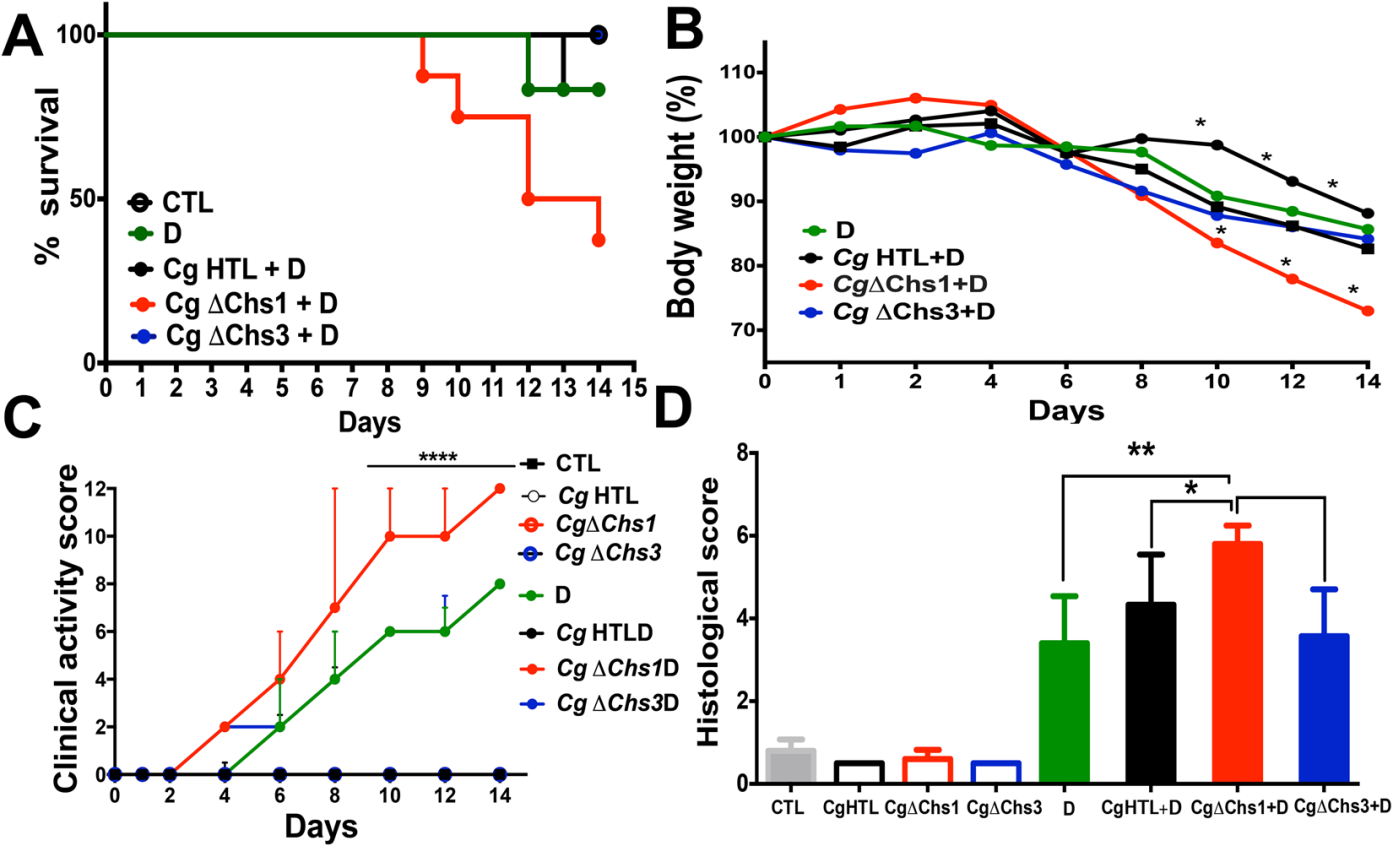
Effect of chitin-deficient *C. glabrata* on inflammatory parameters. (**A**) Mouse survival. Results are expressed as percent survival from the time of *C. glabrata* HTL, *C. glabrata ΔChs1*, or *C. glabrata ΔChs3* challenge and DSS treatment. The survival data were significantly different by the log-rank test (*P* < 0.05). (**B**) Mouse body weight. The data shown are the mean ± SD from two independent experiments. (**C**) Clinical analysis of DSS-induced colitis in mice. ***P < 0.001 for *C. glabrata ΔChs1* + D vs. *C. glabrata ΔChs3* + D and *C. glabrata* HTL + D. (**D**) Histologic scores. Data are the mean ± SD of 16 mice per group (*P < 0.001).

To assess the impact of chitin deficiency on persistence of *C. glabrata* in the gut, *C. glabrata* strains ΔChs1 (Cg ΔChs1), and Δchs3 (Cg ΔChs3) were used in the DSS-induced colitis model and were compared with their parental strain, HTL (Fig. 5). Phenotypically, no growth defect was found in any strain when grown on YPD medium (data not shown). Flow cytometry revealed a significant decrease in chitin levels in *C. glabrata* ΔChs3 (80–90%), while chitin levels were increased in *C. glabrata* ΔChs1 when compared to *C. glabrata* HTL (Supplementary data Fig. 2). Additionally, β-man levels were increased in *C. glabrata* ΔChs1 when compared to *C. glabrata* ΔChs3 or *C. glabrata* HTL (Supplementary data Fig. 2). These data were consistent with confocal microscopy, which revealed that *C. glabrata* ΔChs3 stained weakly with WGA, while *C. glabrata* ΔChs1 and *C. glabrata* HTL stained strongly with WGA, and this staining was particularly strong in *C. glabrata* ΔChs1.

Mice were administered a unique inoculum of these *C. glabrata* strains orally. In the absence of DSS, neither mouse mortality, nor clinical inflammation was observed in groups receiving *C. glabrata* (Fig. 5).

In mice with DSS-induced colitis, mortality rates were recorded as 20% in DSS mice, as 20% in *C. glabrata* HTL-DSS, and as 62.5% in *C. glabrata* ΔChs1 mice. This suggests that *C. glabrata* ΔChs1 is more virulent than the other strains (Fig. 5A).

Significantly higher clinical and histologic scores for inflammation were observed in *C. glabrata ΔChs1*-DSS mice than in the *C. glabrata ΔChs3*-DSS and *C. glabrata* HTL-DSS groups (Fig. 5C,D). In addition, histologic sections of colons from *C. glabrata ΔChs1*-DSS mice revealed high levels of leukocyte infiltration, epithelial damage, and edema when compared to colons from *C. glabrata ΔChs3*-DSS or *C. glabrata* HTL-DSS mice. In terms of fungal colonization, all *C. glabrata* strains were eliminated within 2 days after challenge from mice without colitis. In mice with DSS-induced colitis, there was a significant increase in number of *C. glabrata* colonies detected in stools when the first clinical signs of inflammation appeared. Although *C. glabrata ΔChs1* is highly virulent, the number of CFUs of this strain was lower than that of the *C. glabrata* HTL parental strain (Supplementary data Fig. 3).

To assess the impact of chitin deficiency on *C. glabrata* colonization of the gut, we measured the number of *C. glabrata* CFUs adhering to the stomach, caecum, and colons in the different groups of mice (Supplementary data Fig. 3B).

The number of *C. glabrata ΔChs1* colonies was significantly higher in the stomachs of mice treated with DSS than in *C. glabrata ΔChs3* and *C. glabrata* HTL-DSS treated mice. In terms of fungal dissemination from the gut to the organs, higher numbers of *C. glabrata ΔChs1* colonies were observed while the *C. glabrata ΔChs3* strain was unable to disseminate. A trend towards a large number of *C. glabrata ΔChs1* colonies was observed in the kidneys of DSS-treated mice (Supplementary data Fig. 3C).

The impact of *C. glabrata* colonization on the gut microbiota was also investigated. The change in *E. coli* numbers was determined in freshly collected stool samples (Supplementary data Fig. 4). In contrast to mice not treated with DSS, where the population of *E. coli* remained relatively stable, the *E. coli* population increased significantly in the DSS, *C. glabrata ΔChs1*, and *C. glabrata* HTL groups, as inflammation progressed, when compared to *C. glabrata ΔChs3* mice.

Similarly, *E. faecalis* CFUs were significantly lower in *C. glabrata ΔChs3*-DSS mice than in DSS, *C. glabrata ΔChs1*-DSS, or *C. glabrata* HTL-DSS mice (data not shown). In terms of the activation/expression of receptors and signaling pathways in response to *C. glabrata* sensing and colitis, MBL-C and Myd88 expression increased significantly, while the expression of TLR-4 and PPARγ decreased in the colons in response to *C. glabrata ΔChs3* (Supplementary data Fig. 5).

### Effect of oral administration of chitin on intestinal inflammation, *C. glabrata* overgrowth and the gut microbiota

**Figure 6:**
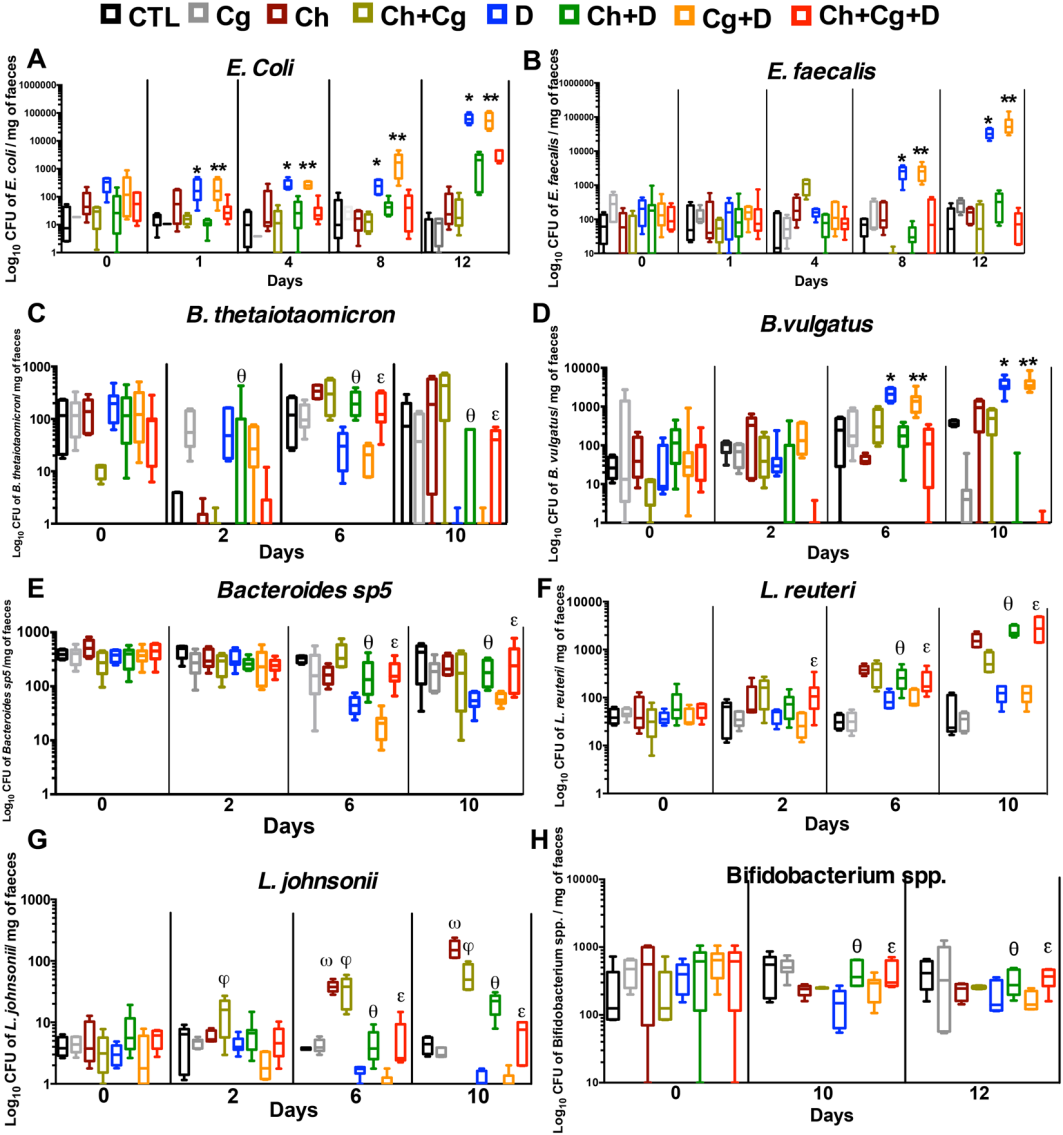
Cultivable bacterial diversity after chitin treatment of mice with DSS-induced colitis. Data are the mean ± SD of 20 mice per group. For all experiments, stool bacteria were isolated from mice on day 0 before *C. glabrata* challenge and DSS treatment. (**A**–**H**) *E. coli, E. faecalis, B. thetaiotaomicron, B. vulgatus, Bacteroides* spp. *TP-5, L. johnsonii, Bifidobacterium* spp., and *L. reuteri* CFUs recovered from stools. Data are the mean ± SD of 16 mice per group (*P < 0.05).

**Figure 7:**
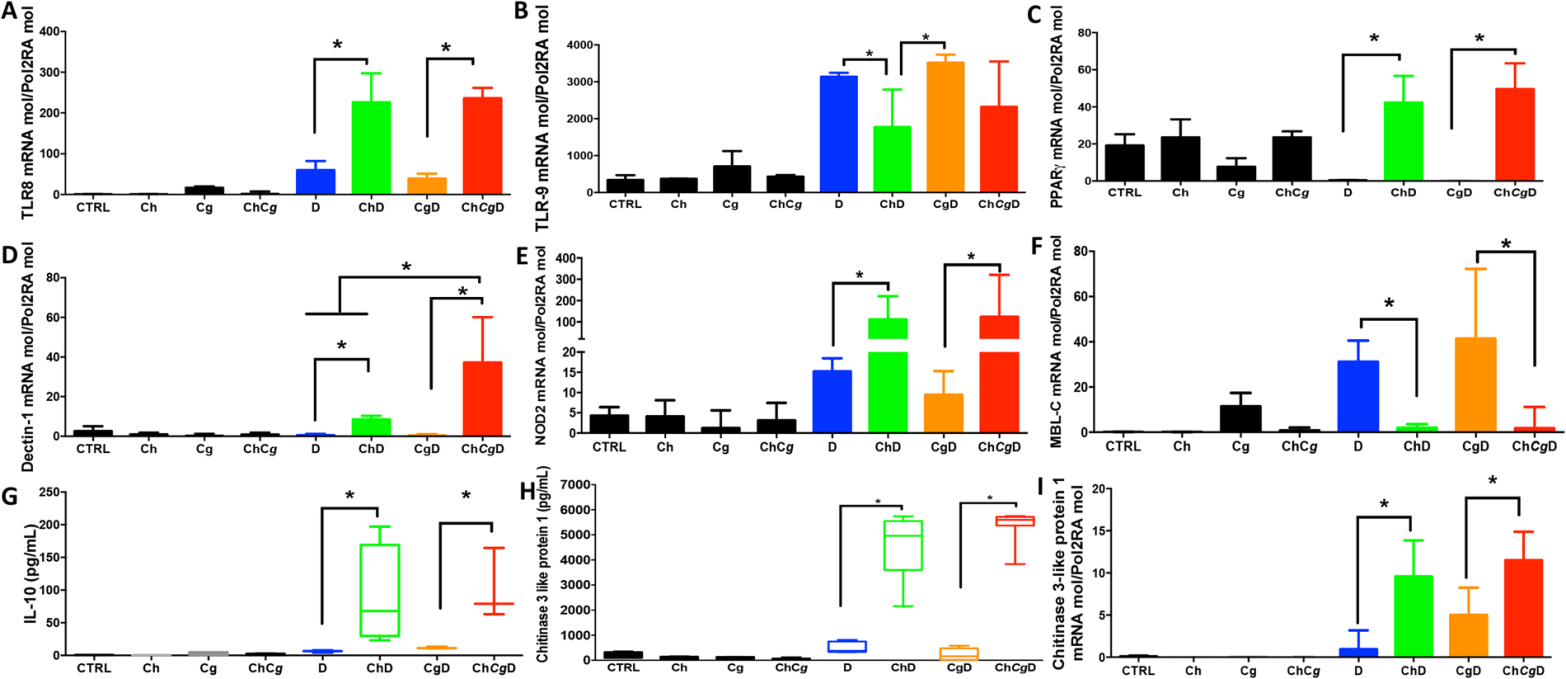
Cytokine and receptor expression after chitin treatment of mice with DSS-induced colitis. (**A**–**F**). Relative expression levels of TLR-8, TLR-9, PPAR-γ, Dectin-1, NOD-2, and MBL-C mRNA in mouse colons. (**G** and **H**) Protein levels of IL-10 and chitinase-3-like-1 protein in mouse colons. (**I**) Relative expression levels of chitinase-3-like-1 protein mRNA in mouse colons. Data are the mean ± SD of 16 mice per group (*P < 0.05).

Before conducting our experiments in mice, we determined the effect of chitin on transepithelial electrical resistance and permeability of Caco-2 cells and whether chitin can reduce the adherence of *C. glabrata* to Caco-2 cells. We used different concentrations of chitin (1, 3, 5, and 10 mg). We found that chitin concentrations greater than 1 mg reduced *C. glabrata* adherence to Caco-2 cells (Supplementary data Fig. 6). Furthermore, the addition of chitin to Caco-2 cells before adding *C. glabrata* promoted intestinal barrier function as measured by a significant increase in transepithelial electrical resistance (data not shown). To determine the effect of chitin on modulation of intestinal inflammation and microbiota diversity including *C. glabrata*, chitin purified from *C. glabrata* was administered orally (3 mg/dose) to mice for 5 days after *C. glabrata* challenge. No mortality was observed in mice that received chitin during DSS-induced inflammation and colonization with *C. glabrata*. Administration of chitin also decreased the clinical and histologic scores when compared to untreated mice (Supplementary data Fig. 7). Mice treated with chitin showed decreased levels of *C. glabrata* in the stools as well as in the stomach and colon (Supplementary data Fig. 8). In terms of bacterial biodiversity, chitin treatment re-established the anaerobic bacteria including *L. reuteri*, *L. johnsonii*, *Bifidobacterium* and *Bacteroides* spp. and reduced aerobic bacteria such as *E. coli* and *E. faecalis* (Fig. 6 and Supplementary data Fig. 9). In addition, TLR-8, dectin-1, NOD-2, and PPARg receptors were significantly activated as a result of chitin administration while the expression of TLR-4, TLR-5, TLR-7, and TLR-9 was decreased (Fig. 7).

When mRNA and protein expression of chitinase-3-like protein-1 was assessed after chitin treatment, a significant increase in chitinase-3-like protein-1 was observed, correlated with a decrease in *C. glabrata* CFUs in the gut indicating that both mRNA and protein expression of chitinase-3 increased after chitin treatment (Fig. 7).

## Discussion

Dysbiosis is a change in the normal gut microbiome with a breakdown of host- microbial crosstalk^20^. This change in the gut microbiome promotes the overgrowth of opportunistic pathogens that contribute to intestinal mucosal inflammation^21,22^. Patients with CD are more frequently colonized with *Candida* species than healthy subjects^23^. Experimental studies show that colonization with *Candida* species exacerbates intestinal inflammation in the DSS-induced colitis model^3,4^. These clinical and experimental observations reveal the major role of opportunistic yeasts in modulation of the host immune-inflammatory response. In the present study, we assessed how the opportunistic yeast *C. glabrata* changes its cell wall composition in order to persist in the gut, and how overgrowth of this fungus together with intestinal inflammation affects the gut microbiome through modulation of both cytokine production and pathogen-recognition receptor (PRR) stimulation. The study also explored the effect of the fungal cell wall component chitin on modulation of the gut microbiome and its biological activities that confer a benefit to the host in terms of reducing inflammation. We also investigated the changes in *C. glabrata* virulence related to chitin gene deletions in the DSS-induced colitis model. In this DSS model, which is used to experimentally mimic IBD, mice received a single inoculum of *C. glabrata* by oral gavage and *C. glabrata* colonization increased gradually when DSS-induced intestinal inflammation was maintained in the mice. Erosions and ulceration of the mucosal surface of the gut promoted overgrowth of *C. glabrata*, which in turn exacerbated inflammation. In contrast, *C. glabrata* was rapidly eliminated from the gut in the absence of DSS-induced colitis, around 2 days after fungal challenge.

In the present study, we observed that regardless of *C. glabrata* colonization, DSS-induced colitis triggered changes in the gut microbiome. We focused in particular on cultivable bacteria belonging to the phyla Firmicutes, Bacteriodetes, Proteabacteria, or Actinobacteria by using selective bacterial media since these bacteria are known to be involved in CD (i.e. *E. coli* and *E. faecalis*)^24^. We observed a decrease in Firmicutes and Bacteroidetes while Proteobacteria and Enterobacteria increased in mice that developed colitis. These data are consistent with the clinical study, which showed a decrease in Firmicutes and Bacteroidetes, and an increase in Proteobacteria and fungal load, in particular *C. glabrata*, in CD patients^25^. Kim *et al*. showed that the dual association of *E. coli* and *E. faecalis*, both commensal organisms, rapidly induced severe pancolitis with dysplasia^26^. In addition, *E. coli* numbers increased during colitis and had a high pro-inflammatory potential to trigger inflammation via TLR-4^27,28^. *E. faecalis* is a facultative anaerobic bacterium that likely benefits from potentially increased oxygen availability in the inflamed intestine, in a manner similar to *E. coli*, which is strictly aerobic. Furthermore, *E. coli* can even exploit the environment in an inflamed gut to obtain a growth advantage when compared to anaerobic bacteria (*Lactobacillus* or *Bifidobacterium*). In response to epithelial cell death and tissue damage during intestinal inflammation, the dead cells could provide extra nutrients, such as ethanolamine, to support *E. coli* overgrowth^29,30^.

Aerobic culture of stomach and colon samples showed an increase in *E. coli* and *E. faecalis* populations in DSS-treated mice when compared to control mice. These data are consistent with clinical studies, which showed that aerobic cultures of biopsies obtained by colonoscopy from control colons were often sterile, whereas colons from patients with CD contained increased bacterial numbers in the sub-mucosa, a relatively well oxygenated site, more than half of which were *E. coli*^31^. In parallel, we observed that the increase in *E. coli* population in the gut was correlated with *C. glabrata* overgrowth. Centeno *et al*. observed that piliated *E. coli* strains can enhance *Candida* attachment to epithelial cells^32^. *E. coli* also exhibits a synergistic effect with *Candida* by inducing high mouse mortality during experimental microbial peritonitis^33^. In biofilm studies, Hoarau *et al*. showed that the interaction of *E. coli* with *C. tropicalis* combined with *Serratia marcescens* enhanced fungal filamentation and biofilm maturation^34^.

In terms of anaerobic bacteria, the population of *Bifidobacterium* spp., *B. thetaiotaomicron* and other *Bacteroides* spp. decreased in response to intestinal inflammation and to *C. glabrata* overgrowth, while *B. vulgatus* was not affected by either DSS-induced colitis or *C. glabrata* overgrowth. Interestingly, *C. glabrata* overgrowth significantly decreased the *L. johnsonii* population during the development of colitis. This observation is consistent with previous reports, which indicate that *Lactobacillus* growth can antagonize colonization by *Candida*^35^.

Receptors and signaling pathways were selected according to our previous studies or other studies showing the involvement of these receptors in fungal recognition, in particular TLRs, MBL, or in the stimulation of signaling pathways in response to *C. glabrata* sensing^7,36,37^. In this study, *C. glabrata* WT was found to increase MBL-C and TLR-9 expression. This finding supports the findings of Choteau *et al*. who showed that the activation of MBL-C by *Candida* sensing in intestinal epithelial cells promoted the rapid elimination of *C. glabrata* from the gut^37^. Conversely, the *C. glabrata* WT strain decreased TLR-4 expression in the mouse colons as colitis developed when compared to colons of mice treated with DSS only, suggesting that stimulation of TLR-4 is driven by overgrowth of *E. coli* and *E. faecalis*, while the increase in MBL-C expression is related to *Candida* sensing^35^. Pro-inflammatory cytokine mRNA and protein expression (IL-6 and IL-1β) increased in the colons of DSS-treated mice, and a higher level of expression of these cytokines was observed in response to *Candida* sensing. This is consistent with different reports, which showed that DSS-induced colitis alone or combined with fungal colonization promotes overexpression of pro-inflammatory mediators that amplify the inflammatory cascade through NF-κB and Myd88 expression.

Previous studies have shown how the local environment affects morphogenesis, virulence gene expression, and stress resistance, but very little is known about how the inflammatory gut environment impacts on the fungal cell wall composition. In the present study, we found that *C. glabrata* undergoes cell wall remodeling during fungal colonization in the DSS-induced colitis model. Modification of the composition of the *C. glabrata* cell wall is related to intestinal inflammation and not to bacteria biodiversity changes, since the *C. glabrata* cell wall modification appears in the days corresponding to the onset of inflammation and not while the bacterial population changes. In the absence of colitis development, the level of α-mans increased in the *C. glabrata* cell wall on days 1 and 2 after fungal challenge and then this yeast was eliminated rapidly from the gut. The increase in *C. glabrata* α-man level enables *C. glabrata* to behave like *S. cerevisiae* transiting through the mouse gut. In contrast, in DSS-induced colitis, *C. glabrata* appears to alter towards a pathogenic form close to that of *C. albicans*, resulting in an increase in chitin and β-man, and a decrease in α-man levels. These data reveal that the inflammatory gut environment impacts on the *C. glabrata* cell wall leading to adaptation of the fungal cell within the host, which allows *C. glabrata* to persist in the gut environment.

In a previous study, we showed that a deficiency in β-mans reduced *C. glabrata* adherence to intestinal epithelial cells, favoring fungal elimination from the mouse gut, indicating that β-mans contribute to *C. glabrata* virulence. However, the role of chitin in modulation of intestinal inflammation, the gut microbiome and fungal colonization has not yet been thoroughly investigated. In the present study, orally administered chitin purified from *C. glabrata* decreased intestinal inflammation and *C. glabrata* overgrowth. Several studies have shown that chitin enhances the immune response and increases the clearance of pathogenic bacteria in animal models; this supports our observations that chitin from *C. glabrata* has beneficial activities for *C. glabrata*.

To assess the impact of chitin deficiency on *C. glabrata* in the inflammatory gut environment, we selected the two genes that encode chitin synthase involved in chitin biosynthesis, *Chs1* and *Chs3*^38^. Flow cytometry and confocal microscopy of the fungal cell wall revealed a significant reduction in chitin in *C. glabrata Δchs3* while chitin levels in *C. glabrata Δchs1* increased, indicating that in contrast to Chs1, chitin synthase Chs3 is a crucial enzyme for cell wall chitin synthesis (Supplementary data Fig. 8). This observation is consistent with other reports that show that chitin synthase Chs3 is involved in generating 80–90% of the total fungal chitin, while Chs1 is responsible for repairing the septa and the weakened cell walls of daughter cells after their separation from mother cells^39^. In the absence of DSS, mutant strains did not induce inflammation in the mice. In DSS-induced colitis, *C. glabrata Δchs1*, rich in chitin and β-glucan, is highly virulent in terms of colonization, fungal dissemination to the organs and inflammatory parameters. In contrast, a deficiency of chitin synthase Chs3 reduced the pathogenicity of *C. glabrata* in the inflammatory gut environment. Furthermore, *C. glabrata Δ* chs1, which has a high level of chitin, does not induce the expression of chitinase-3-like protein-1. Thus, this promotes *C. glabrata* overgrowth and persistence in the gut. In addition to the increase in chitin level in the *C. glabrata Δ*chs1 cell wall, the β-man level, which is expressed in the outer fungal cell wall layer, was also increased. These data are consistent with other reports, which show that *C. glabrata* strains deficient in β-mans are less virulent than the WT strain suggesting the involvement of β-mans in the virulence and resistance of *C. glabrata* in the intestinal tract^5^.

Interestingly, the virulent *C. glabrata Δchs1* strain reduced the expression of MBL-C, TLR-2, and TLR-9, while *C. glabrata Δchs3* increased the expression of these receptors as colitis developed, indicating that the cell wall of *C. glabrata Δchs3* is rich in α-mans, which are potential ligands for MBL-C and TLR4. In contrast, *C. glabrata Δchs1*, which has high levels of expression of β-man epitopes on its cell wall surface, is capable of reducing these intestinal epithelial cell receptors as an escape mechanism from the host defense.

Chitin acts as an anti-inflammatory agent and is involved in the process of wound healing^40^. Oral administration of chitin reduced all of the inflammatory parameters, and led to overgrowth of *C. glabrata* and reestablishment of the biodiversity of the gut microbiota. Chitin treatment increased chitinase-3-like protein-1, promoting chitin breakdown and the generation of small sized chitin particles that induce IL-10 production via PPARg, NOD-2, and TLR-8 sensing. These results are consistent with those of Wagner *et al*. who showed that chitin oligosaccharides have the potential to induce IL-10 secretion, via NOD-2 and TLR-9 signaling, promoting the attenuation of inflammation responses^19^. Surprisingly, the expression levels of TLR-4, TLR-5, TLR-6, TLR-7, TLR-9, and MBL-C were not upregulated by chitin treatment.

In conclusion, inflammation in the gut increased the aerobic bacteria population, in particular *E. coli* and *E. faecalis*, but decreased the population of anaerobic bacteria such as *L. johnsonii*, *B. thetaiotaomicron*, and *Bifidobacterium* spp. DSS-induced colitis led to cell wall remodeling through an increase in chitin production, which was involved in promoting *C. glabrata* overgrowth and persistence in the gut. *C. glabrata* colonization modulated the intestinal epithelial receptors, in particular MBL-C, TLR-4, and TLR-9, as well as expression of the signaling pathways, NF-κB and Myd88. In terms of fungal cell wall components, oral administration of chitin to mice reduced the overgrowth of aerobic bacteria and *C. glabrata* as well the production of inflammatory parameters through stimulation of intestinal receptors. Chitin deficiency affected the *C. glabrata* cell wall composition. This deficiency was compensated for by an increase in α-man levels in *C. glabrata chs3*, which induced fewer inflammatory parameters than the parental strain. Chitin treatment increased chitinase-3-like protein-1, enabling chitin digestion and the generation of small sized chitin particles that induced IL-10 production via PPARg, NOD-2, and TLR-8 sensing, promoting the attenuation of colitis and *C. glabrata* elimination. Finally, this study has increased our understanding of the nature of yeast molecular components that differentially affect inflammation and/or *C. glabrata* clearance.

## Methods

### Animals

Eight-to-10-week-old, female *C57BL*/*6* mice were purchased from Charles River Laboratories, France. Mice were allocated to six experimental groups (Supplementary data, Table 1) and eight control groups, including assessment of the effect of DSS alone. Four complete experimental series were performed independently.

All experimental procedures were approved by the subcommittee for Research Animal Care, Regional Hospital Center, Lille, France (00550.05), and in accordance with institutional (86/609/CEE) and European guidelines for the care and use of laboratory animals.

### Yeast strains

The *C. glabrata* strains used are shown in Supplementary data Table 2. For *C. glabrata* ΔChs3, CAGL0B04389g CHS3 was deleted (alias CHS3A)^38^. All *C. glabrata* strains were maintained at 4 °C in yeast peptone dextrose broth (YPD; 1% yeast extract, 2% peptone, 2% dextrose).

### Extraction of chitin from *C. glabrata*

*C. glabrata* cell pellets were washed twice in phosphate-buffered saline (PBS). Chitin was extracted from *C. glabrata* yeast cells as described previously^41^. Briefly, the cell pellet was incubated twice in 20 ml of 10% KOH and autoclaved at 120 °C for 2 h. After washing several times with distilled water, the supernatant was removed and the pellet was oxidized with 50% hydrogen peroxide and 50% acetic acid, and then autoclaved at 120 °C for 2 h. After washing several times with distilled water, the chitin fraction was lyophilized. The nature of the chitin was confirmed by nuclear magnetic resonance (NMR) analysis. Intact chitin purified from *C. glabrata* was suspended in deuterated hexafluroisopropanol (Euriso-Top) at 70 °C until dissolved. All experiments were performed using a Bruker Avance 600 MHz (13.1 T) spectrometer with Bruker standard pulse programs. The chitin concentration was determined using a BiCinchoninic acid assay. The standard range for the N-acetyl-glucosamine (GlcNAc) control was 0.1–5 mg/mL.

### Measurement of cell wall chitin content

The chitin content was measured as described previously^42^. *C. glabrata* HTL, *C. glabrata ΔChs1*, and *C. glabrata ΔChs3* strains were grown in YPD broth. Yeast cells were suspended in 10 mL of PBS to a final concentration of 10^9^ cells/mL and were disrupted with glass beads. The cells were then washed several times with 1 M NaCl and extracted in SDS-MerOH buffer (50 mM Tris, 2% sodium dodecyl sulfate (SDS), 0.3 M β-mercaptoethanol, 1 mM EDTA; pH 8.0) at 100 °C for 10 min, and then washed in distilled water. The chitin concentration was determined using a BiCinchoninic acid assay. The standard range for the N-acetyl-glucosamine (GlcNAc) control was 0.1–5 mg/mL.

### Inoculum preparation and induction of colitis

The mice were inoculated on day 1 by oral gavage with 200 µL PBS containing 5 × 10^7^ live *C. glabrata* cells. From day 1 to day 14, mice were also administered 2% DSS (36−50 kDa; MP Biomedicals, LLC, Germany) in drinking water in order to induce intestinal inflammation. For chitin treatment, mice were administered with chitin purified from *C. glabrata* (3 mg per mouse) orally and daily for 5 days, starting on day 1. The presence of *C. glabrata* in the intestinal tract was monitored daily by measuring the number of colony-forming units (CFUs) in feces (approximately 0.1 g/sample) collected from each animal^4^. Fecal samples were suspended in 1 mL saline, homogenized in a glass tissue homogenizer, and samples were then cultured on Candi-Select medium (Bio-Rad Laboratories, Marnes la Coquette, France)^43^. The number of *C. glabrata* colonies was counted after incubation of the plates at 37 °C for 48 h. The results are expressed as CFU/µg of feces. In order to determine the degree of *C. glabrata* colonization of the gut, the mice were sacrificed and the GI tract was removed and separated into the stomach, ileum, and colon. These portions of the GI tract were cut longitudinally and the intestinal contents were removed. The tissue samples were then washed several times in PBS in order to minimize contamination from yeasts present in the lumen^44^. Serial dilutions of the homogenates were prepared and plated onto Candi-Select medium. The number of colonies was noted after 48 h incubation at 37 °C and expressed as *C. glabrata* CFU/mg of tissue.

For the isolation of bacteria, we performed serial dilutions of the gut contents (stomach, colon, and caecum) or fecal samples collected from the mice. The samples were cultured on non-selective bacterial media (AC agar) focusing on the most representative cultivable anaerobic and aerobic bacteria that can undergo changes during intestinal inflammation^45^.

For the isolation of aerobic bacteria, the fecal samples and tissues were plated onto MacConkey agar (Sigma-Aldrich), bile esculin azide agar (BEA; Sigma-Aldrich), and MRS agar (Sigma-Aldrich) plates. Serial dilutions of the samples were prepared. Bacteroides bile esculin (BBE; Sigma-Aldrich), Columbia agar (Sigma-Aldrich), and Bifidus selective medium agar (BSM; Sigma-Aldrich) were used for the isolation of anaerobic bacteria. These plates were incubated in anaerobic jars containing an anaerobic atmosphere generator pack (AnaeroGen^TM^ 2.5 L; Sigma-Aldrich) at 35 °C. All aerobic and anaerobic media contained 60 mg/L fluconazole (Fresenius Kabi) to inhibit the growth of fungal cells.

All plates were incubated at 37 °C and examined after 24 h and 48 h. To identify any bacteria on the plates, the colonies were mixed with 1.5 μl of matrix solution (α-cyano-4-hydroxycinnamic acid; Bruker Daltonics) dissolved in 50% acetonitrile, 47.5% water, and 2.5% trifluoroacetic acid, and analyzed by MALDI-TOF MS (Microflex-Bruker Daltonics).

### Analysis of *C. glabrata* cell wall remodeling after passage through the GI tract

Fresh fungal cells obtained from fecal samples, and collected daily from each tagged animal, were diluted in PBS. 10^7^ *C. glabrata* cells were prepared and washed several times with PBS. Washed yeast cells were incubated with PBS containing 2% fetal calf serum for 20 min, and were then incubated with fluorescein isothiocyanate-wheat germ agglutinin (FITC-WGA), rhodamine-labeled concanavalin A (Con A), or 5B2 antibody (FITC anti-rat IgM secondary antibody and the control isotype rat IgM). Analysis of the expression of glycan epitopes on the *C. glabrata* cell wall was performed by flow cytometry (Accuri^®^ Sampler™). Mean fluorescence intensity (MFI) of each histogram was calculated as: labeled strain-unlabeled strain/mean fluorescence of labeled strain according to the number of days.

### Assessment of clinical and histological scores

Body weight and mortality of the mice were recorded daily. The data were expressed as mean percent change from initial body weight. Clinical scores ranging from 0 to 12 and histologic scores ranging from 0 (no changes) to 6 (extensive cell infiltration and tissue damage) were calculated as described previously^4,46^.

### Real-time mRNA quantification of pro-inflammatory cytokines and innate immune receptors

Total RNA was extracted from mouse colons using a Nucleospin RNA kit (Macherey-Nagel). RNA was quantified by spectrophotometry (Nanodrop; Nyxor Biotech, France). Reverse transcription of mRNA was carried out from 1 µg total RNA using a high capacity cDNA RT kit (Applied Biosystems) in a final volume of 20 µL. cDNA was amplified by PCR in the one-step system (Applied Biosystems) using Fast SYBR green (Applied Biosystems). The intensity of SYBR green dye was assessed using one-step software. The reference gene, *POLR2A*^47^, was used to normalize the results.

### Quantification of cytokines and chitinase 3-like 1 by ELISA

Representative pro-inflammatory (IL-1β, IL-6) and anti-inflammatory (IL-10) cytokine profiles were selected in this study. Cytokine concentrations (IL-1β, IL-6, and IL-10) in the colons were measured using a commercial ELISA kit according to the manufacturer’s instructions (eBioscience, San Diego, CA) whereas the detection of murine chitinase 3-like 1 levels was done using an ELISA kit from R and D systems. The data are expressed as pg/mL.

### Statistical analysis

All data are expressed as the mean ± standard deviation (SD) for each experimental group. Pairs of groups were compared using the Mann-Whitney U test. Differences were considered to be statistically significant when the P value was as follows: p < 0.05; p < 0.01; p < 0.001.

All statistical analyses were carried out using GraphPad Prism 4.0 and XLSTAT.

## Electronic supplementary material

Supplementary Information
