# Supplementary material for: Remodeling of the Candida glabrata cell wall in the gastrointestinal tract affects the gut microbiota and the immune response
Source: Sci Rep. 2018 Feb 20;8:3316. doi: 10.1038/s41598-018-21422-w (PMC5820338; doi:10.1038/s41598-018-21422-w)
Supplement: Supplementary file 1 — Supplementary Information [file 41598_2018_21422_MOESM1_ESM.docx]

**Remodeling of the *Candida glabrata* cell wall in the gastrointestinal tract affects the gut microbiota and the immune response**

Rogatien Charlet, Youri Pruvost, Gael Tumba, Fabian Istel, Daniel Poulain, Karl Kuchler, Boualem Sendid, Samir Jawhara

**Supplementary data**


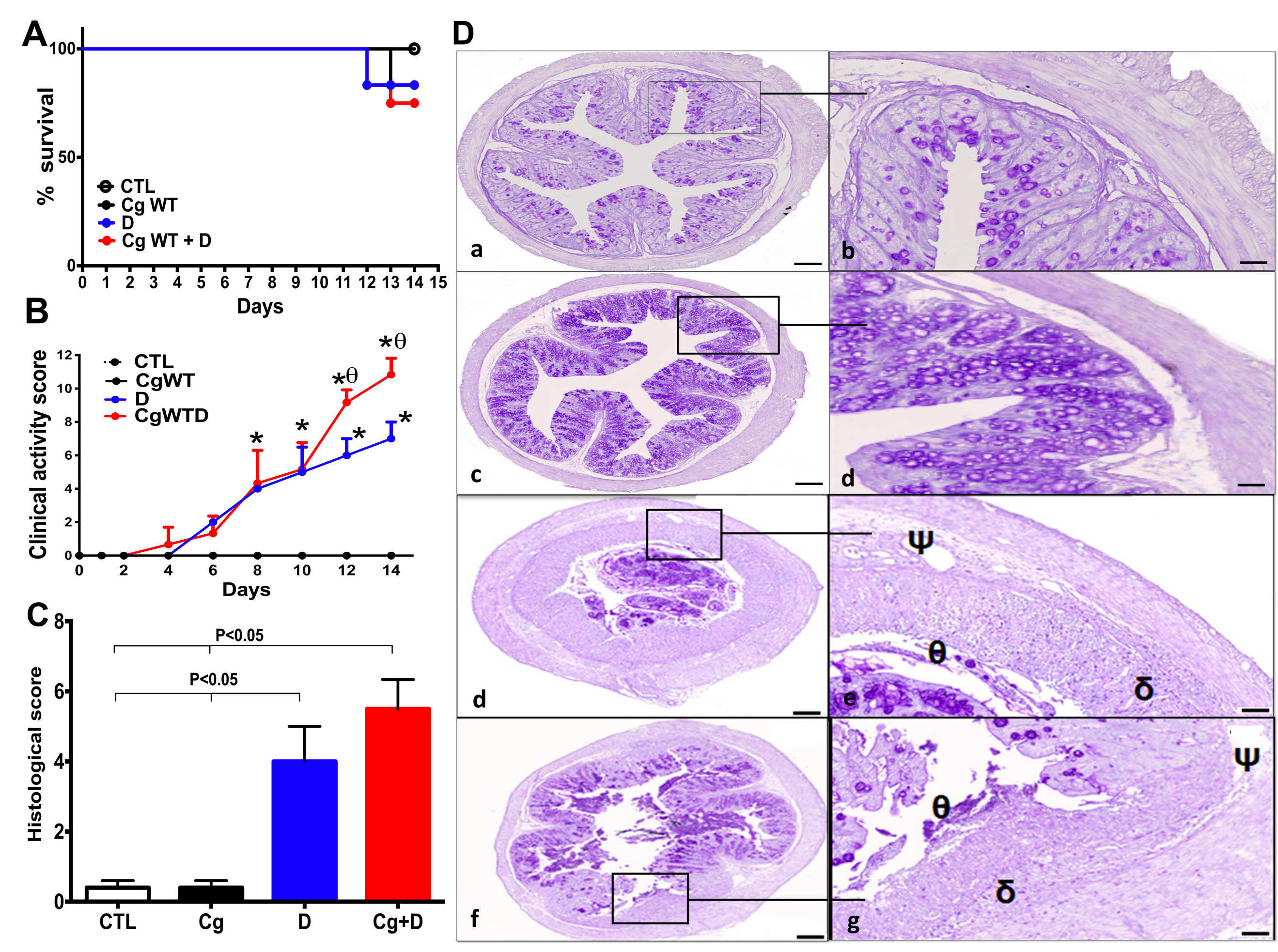


**Figure 1: Effect of *C. glabrata* wild-type (WT) colonization on DSS-induced colitis. (a) Mouse survival.** Results are expressed as percent survival from the time of *C. glabrata* WT challenge and DSS treatment. No mouse mortality was recorded in the CTL and CgWT groups. This led to overlapping of the two curves. The survival data were significantly different by the log-rank test (*P*<0.05). **(b)** **Clinical analysis of DSS-induced colitis in mice.** Clinical score was determined by assessing weight loss, change in stool consistency, and presence of gross bleeding. The CTL and CgWT groups did not show any signs of inflammation since *C. glabrata* was rapidly eliminated from the gut. Thus, the curves for CTL and CgWT overlapped indicating the absence of clinical activity score for these two groups. The clinical score ranged from 0 to 12 (each value corresponds to the mean value for 14 days per group). **P*<0.05 for DSS (D) and *C. glabrata* WT+DSS (*Cg*WTD) mice vs. control (CTL) and *C. glabrata* WT (*Cg*WT) mice. ^θ^*P*<0.05 for *C. glabrata* WT+DSS (*Cg*WTD) mice vs. DSS (D) mice. **(c) Histologic scores**. Mice were exposed to 2% DSS in drinking water for 14 days. Scores range from 0 (no changes) to 6 (extensive cell infiltration and tissue damage). Data are the mean ± SD of 20 mice per group (*P*<0.001). **(d)** **Histologic analysis of the colon in *C. glabrata* and DSS-induced colitis.** Panels *Cg*WT correspond to colon sections from WT mice receiving *C. glabrata* WT only (*Cg*). Panels DSS correspond to colon sections from mice receiving DSS. Panels *Cg*WTDSS correspond to colon sections from mice receiving *C. glabrata* and DSS. In the absence of DSS, no significant differences in the colon sections were observed between control animals (not inoculated) and those that received *C. glabrata*. The colon sections from DSS mice show an inflammatory cell infiltrate in the colon wall structures. The colon sections from *Cg*WTD mice show a high inflammatory cell infiltrate in the colon wall structures and massive tissue destruction (asterisk,). The scale bars represent 50 µm (a, c, e, g, i, and k) and 10 µm (b, d, f, h, j, and l).


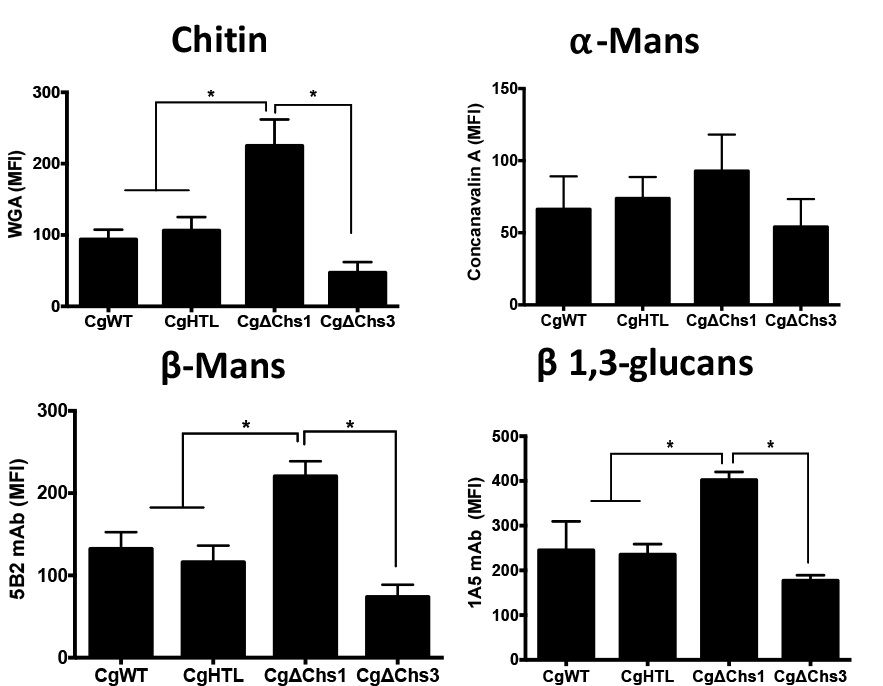


**Figure 2: Flow cytometry analysis of cell wall surface glycan expression in *C. glabrata*.** Cell wall surface glycan expression was analyzed in *C. glabrata* wild-type (WT), *C. glabrata* HTL, *C. glabrata* ∆chs1, and *C. glabrata* ∆chs3 using WGA, concanavalin A, mAbs 5B2 and 1A5 immunofluorescent staining.

**
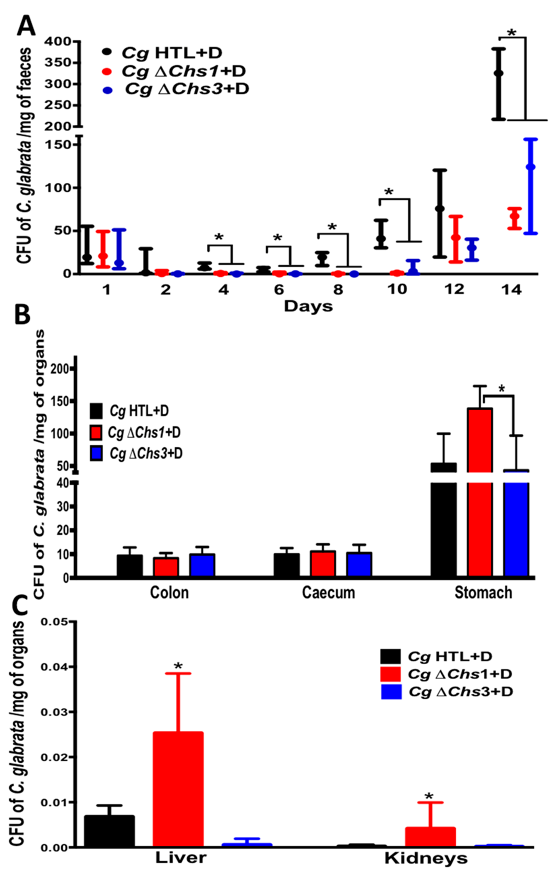
**

**Figure 3: Impact of chitin deficiency on *C. glabrata* colonization in mouse DSS-induced colitis. (a) Number of *C. glabrata* colony forming units (CFU) recovered from stools.** Data are the mean ± SD of 20 mice per group. **(b) Number of *C. glabrata* CFU recovered from the stomach, caecum, and colon.** Data are the mean ± SD of 20 mice per group (**P*<0.001). **(c) Number of *C. glabrata* CFU recovered from the liver and kidneys.** Data are the mean ± SD of 20 mice per group (**P*<0.001).

**
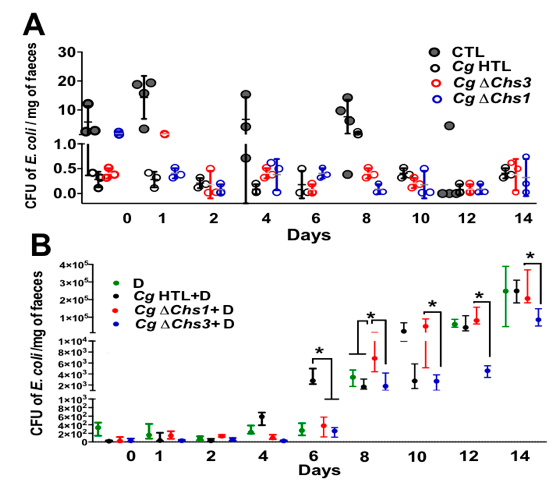
**

**Figure 4: Effect of chitin-deficient *C. glabrata* on *E. coli* overgrowth in DSS-induced colitis*.* (a)** Four groups consisted of controls (water), *C. glabrata* HTL alone (*Cg* HTL), *C. glabrata* *ΔChs1* (*Cg* *ΔChs1*), and *C. glabrata* *ΔChs3* (*Cg* *ΔChs3*). Data are the mean ± SD of 20 mice per group. **(b)** Four groups consisted of DSS (D), *C. glabrata* HTL+DSS (*Cg* HTL+D), *C. glabrata* *ΔChs1*+DSS (*Cg ΔChs1*+D), and *C. glabrata* ΔChs3+DSS (*Cg* *ΔChs3*+D). Data are the mean ± SD of 20 mice per group. For all experiments, stool bacteria were isolated from mice on day 0 before *C. glabrata* challenge and DSS treatment. Data are the mean ± SD of 20 mice per group (**P*<0.05).

**
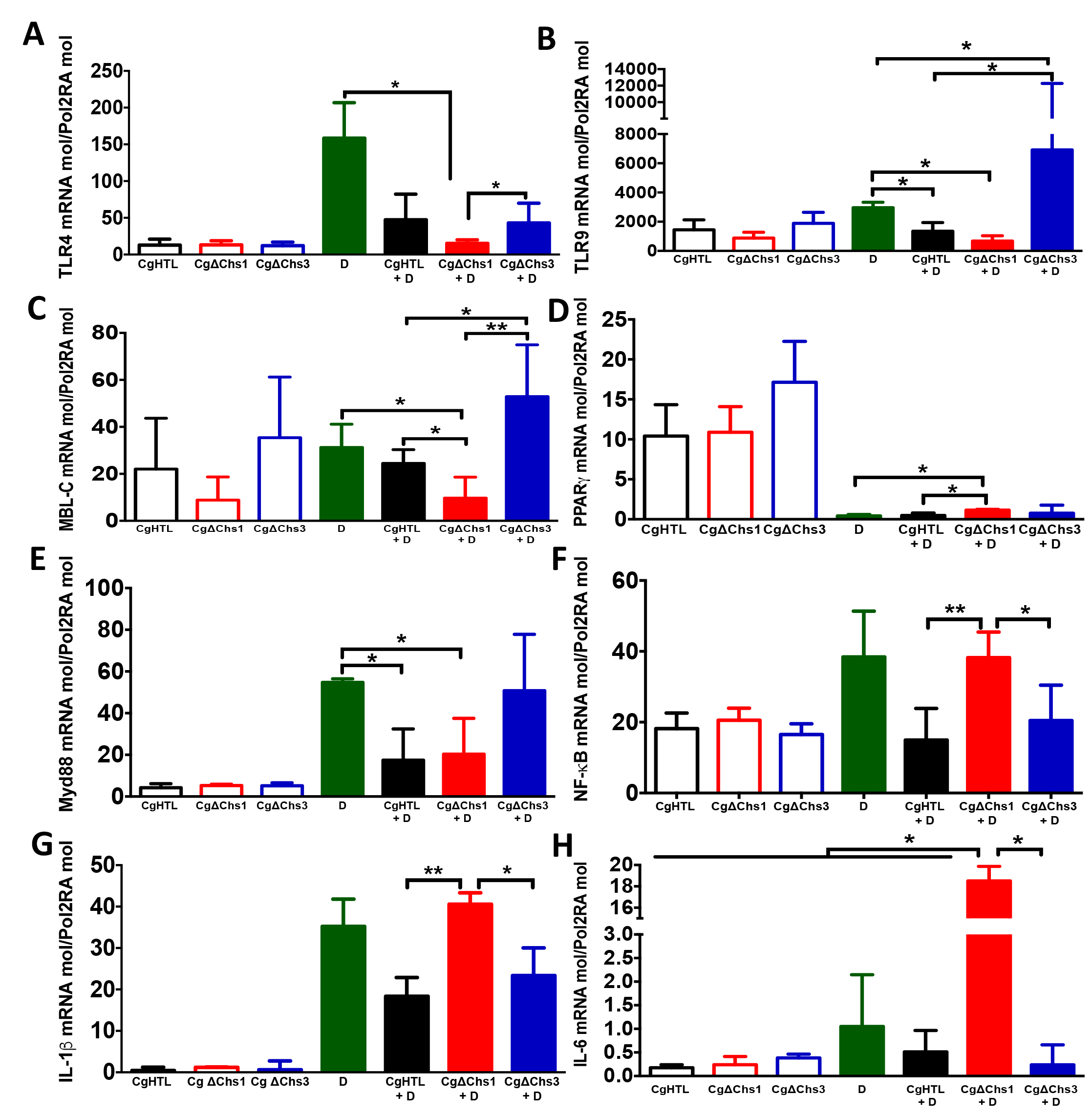
**

**Figure 5: Modulation of receptor and cytokine expression in mice with DSS-induced colitis**. **(A, B, C and D)** **Relative expression levels of TLR-4, TLR-9, MBL-C and IL-10 mRNA in mouse colons**. Data are the mean ± SD of 20 mice per group (**P*<0.05).

**
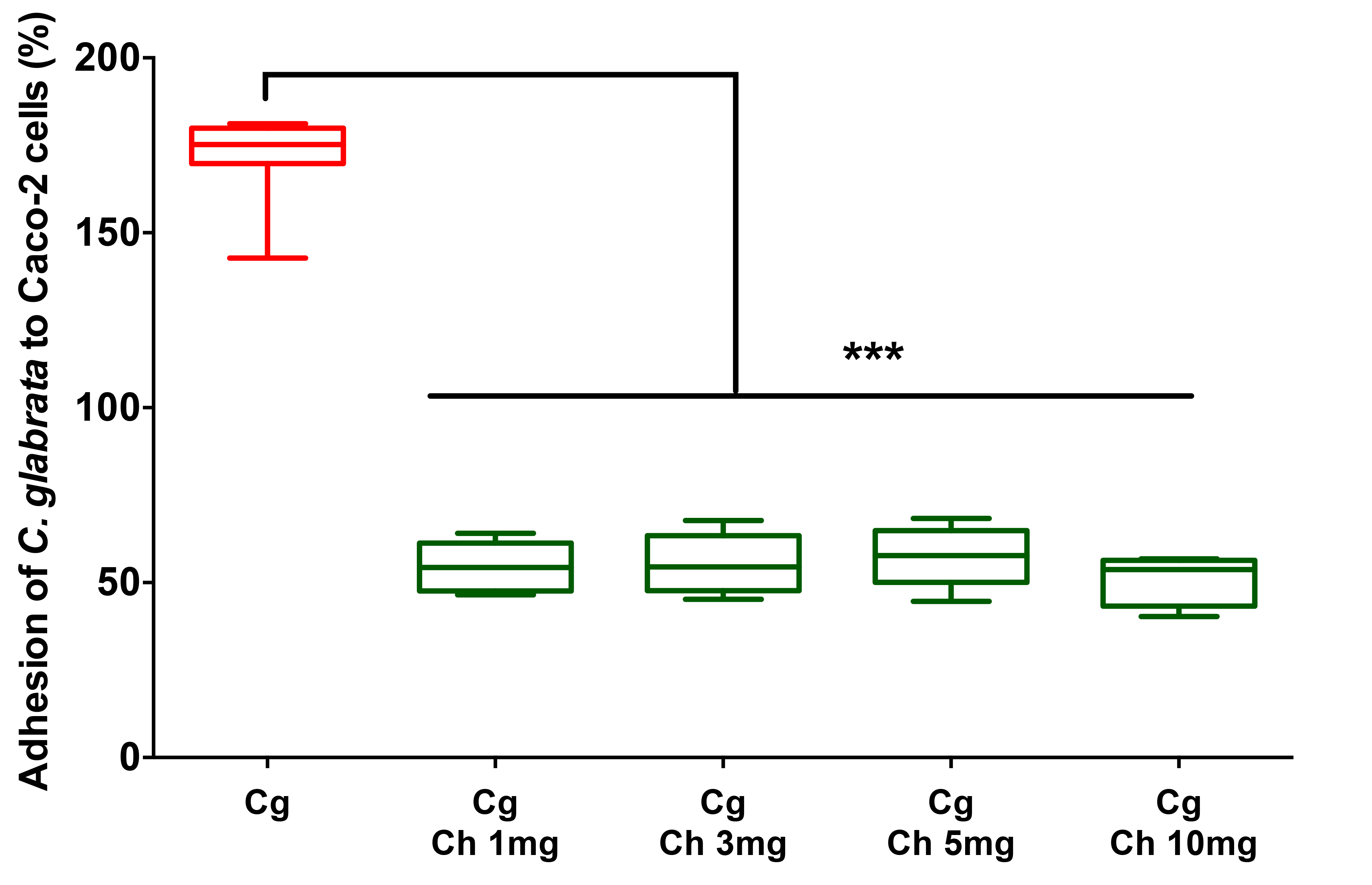
**

**Figure 6: *C. glabrata* adhesion to Caco-2 cells.** 10^5^ *C. glabrata* cells were labelled with calcein and added to 5 × 10^5^ Caco-2 cells treated or not with a different concentration of chitin (1, 3, 5 or 10mg). Cg corresponds to the adhesion of *C. glabrata* to Caco-2 cells while Cg+Ch corresponds to the adhesion of *C. glabrata* to Caco-2 cells treated with a different concentration of chitin. Results are expressed as percent *C. glabrata* adhering to Caco-2 cells.

**Figure 7: Impact of chitin treatment on inflammatory parameters. (a) Mouse survival. (A)** **Clinical analysis of DSS-induced colitis in mice. (B) Histologic scores**. Data are the mean ± SD of 20 mice per group (**P*<0.001). **(C)** **Histologic analysis of the colon.** Panels a and b correspond to colon sections from wild-type (WT) mice receiving either chitin or *C. glabrata* WT + chitin (*Cg*). Panels c and d correspond to colon sections from mice receiving chitin and DSS. Panels e and f correspond to colon sections from mice receiving chitin, *C. glabrata* and DSS. In the absence of DSS, no significant differences in the colon sections were observed between control animals (not inoculated) and those that received *C. glabrata*. The scale bars represent 50 µm (a, b, c, and e) and 10 µm (d, and f).

**
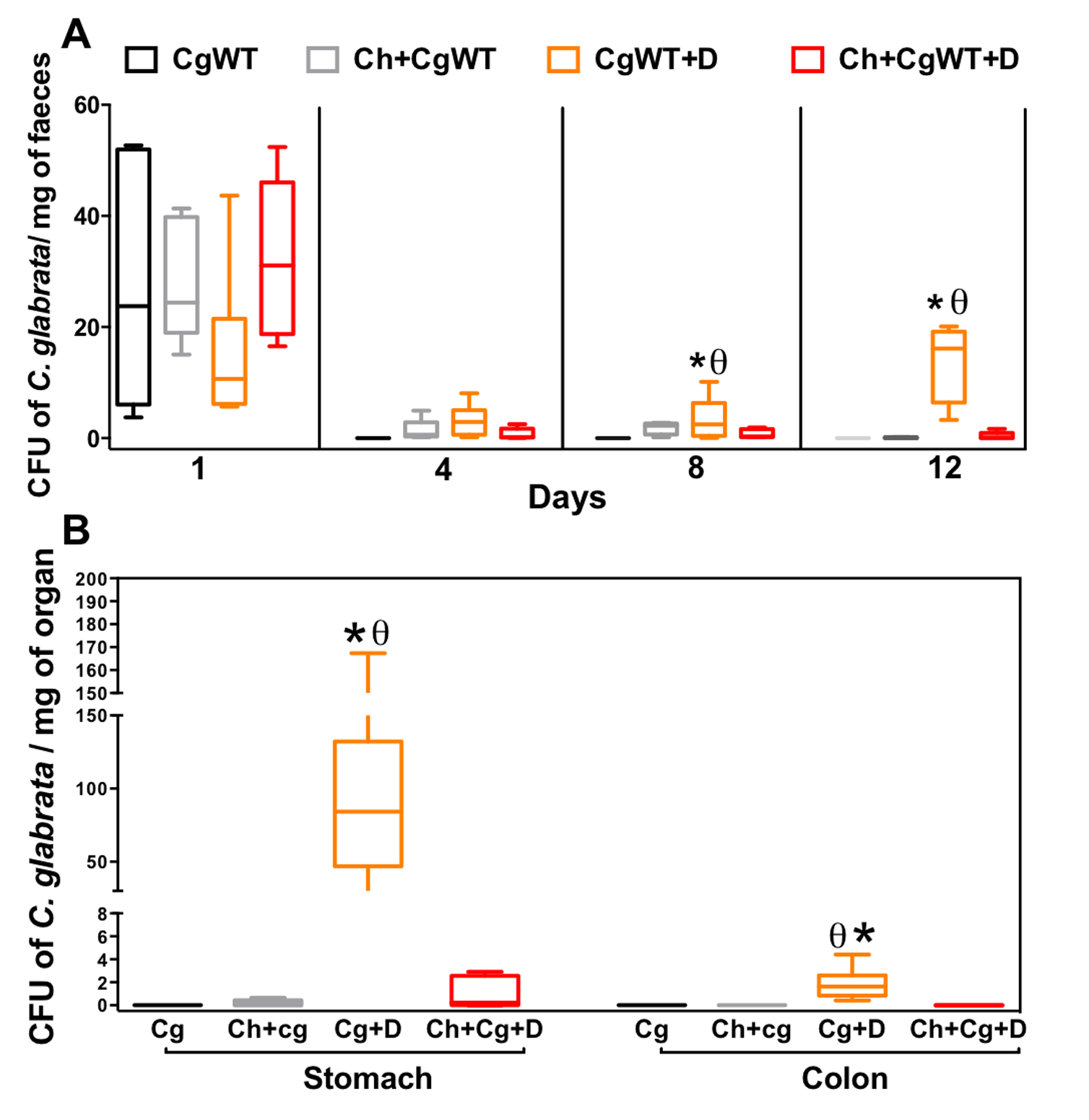
**

**Figure 8: Effect of chitin treatment on elimination of *C. glabrata* from the gut. (a) Number of *C. glabrata* colony forming units (CFU) recovered from stools.** Data are the mean ± SD of 20 mice per group. **(b) Number of *C. glabrata* CFU recovered from the stomach, and colon.** Data are the mean ± SD of 20 mice per group (**P*<0.001).

**
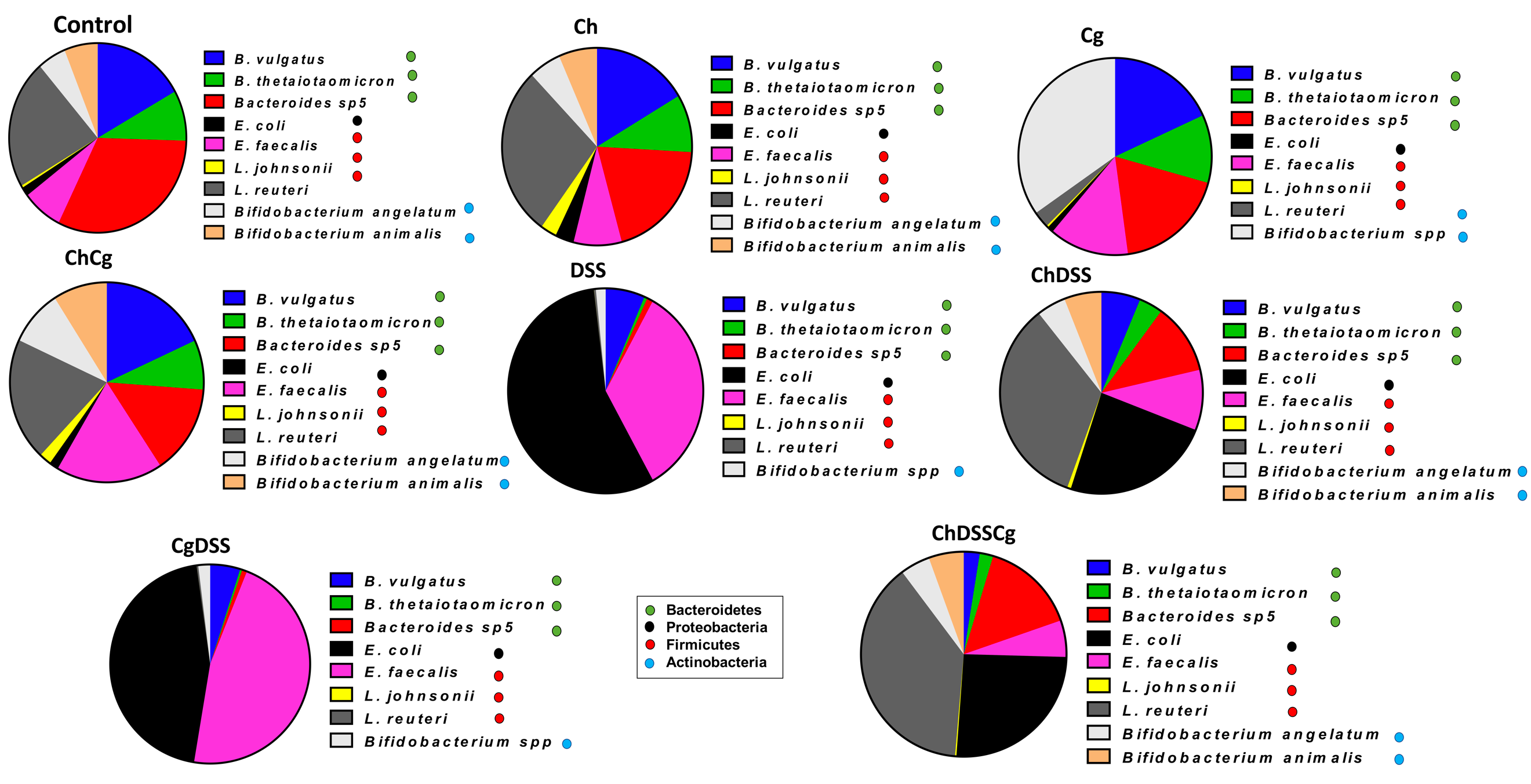
**

**Figure 9: Summary of the effects of oral chitin administration on modulation of the cultivable microbiota biodiversity in *C. glabrata* DSS-treated mice.**

**Table 1: Experimental mouse groups and percent survival of the mice.**

| Group | Inoculum and DSS treatment | N^a^ | Survival (%) |
| --- | --- | --- | --- |
| CTL | Water | 10 | 100 |
| CgWT | *C. glabrata* WT | 10 | 100 |
| Ch | chitin | 10 | 100 |
| CgWT+Ch | *C. glabrata* WT+ chitin | 10 | 100 |
| CgHTL | *C. glabrata HTL* | 10 | 100 |
| Cgchs1Δ | *C. glabrata chs1 Δ* | 10 | 100 |
| Cgchs3Δ | *C. glabrata chs3 Δ* | 10 | 100 |
| D | DSS | 16 | 90 |
| D+Ch | DSS+chitin | 16 | 100 |
| CgWT+DSS | *C. glabrata* WT+ DSS | 16 | 80 |
| CgWT+DSS+Ch | *C. glabrata* WT+ DSS+chitin | 16 | 100 |
| CgHTL+DSS | *C. glabrata* HTL+ DSS | 16 | 90 |
| Cgchs1Δ+D | *C. glabrata chs1 Δ +*DSS | 16 | 37.5 |
| Cgchs3Δ+D | *C. glabrata chs3 Δ +*DSS | 16 | 100 |

**Table 2: *C. glabrata* strains used in the study.**

| **Strain** | **Description** | **Parent strain** | **Genotype** |
| --- | --- | --- | --- |
| *C. glabrata ATCC 2001* | Wild-type |  | MATa^1^ |
| *C. glabrata* HTL | his::FRTleu2Δ::FRTtrp1Δ::FRT | *C. glabrata* ATCC 2001 | ^2^ |
| *C. glabrata chs1Δ* | Δchs1 | *C. glabrata* HTL | ^2^ |
| *C. glabrata chs3Δ* | Δchs3 | *C. glabrata* HTL | ^2^ |

**References**

1 Dujon, B. *et al.* Genome evolution in yeasts. *Nature* **430**, 35-44, doi:10.1038/nature02579 (2004).

2 Schwarzmuller, T. *et al.* Systematic phenotyping of a large-scale Candida glabrata deletion collection reveals novel antifungal tolerance genes. *PLoS pathogens* **10**, e1004211, doi:10.1371/journal.ppat.1004211 (2014).
